# Monocyte programmed death ligand-1 expression is an early marker for predicting infectious complications in acute pancreatitis

**DOI:** 10.1186/s13054-017-1781-3

**Published:** 2017-07-14

**Authors:** Tingting Pan, Tianyun Zhou, Lei Li, Zhaojun Liu, Ying Chen, Enqiang Mao, Meiling Li, Hongping Qu, Jialin Liu

**Affiliations:** 1 Department of Critical Care Medicine, Ruijin Hospital, Shanghai Jiaotong University School of Medicine, 197 Rui-Jin Er Road, Shanghai, 200025 China; 2 Department of Emergency Intensive Care Unit, Ruijin Hospital, Shanghai Jiaotong University School of Medicine, Shanghai, China

**Keywords:** PD-L1, Acute pancreatitis, Infection, Immunosuppression, Prediction

## Abstract

**Background:**

Acute pancreatitis (AP) is a life-threatening disease that requires early identification of patients at risk of developing infectious complications. Immunosuppression is an initial event that is key to AP pathogenesis. The programmed cell death 1 (PD-1) and programmed cell death ligand 1 (PD-L1) system is reported to mediate evasion of host immune surveillance in many diseases; however, the relationship between PD-1/PD-L1 expression and these parameters or infectious complications in AP has not been elucidated. This study was conducted to determine whether PD-1 and PD-L1 are upregulated and to reveal the relationship between PD-1/PD-L1 expression and the development of infectious complications in AP.

**Methods:**

Sixty-three patients with AP and 32 sex- and age-matched healthy control subjects were prospectively enrolled. On days 1 and 3 after the onset of AP, we measured PD-1 expression in peripheral CD4^+^ T cells and PD-L1 and human leukocyte antigen-DR (HLA-DR) expression in CD14^+^ monocytes using flow cytometry. Plasma interleukin (IL)-10 levels were measured by enzyme-linked immunosorbent assay.

**Results:**

Compared with healthy volunteers, the percentages of PD-1-expressing CD4^+^ lymphocytes and PD-L1-expressing CD14^+^ monocytes were increased in patients with AP on days 1 and 3 after onset, especially those with infectious complications. Moreover, increased PD-1/PD-L1 expression was associated with increased occurrence of infectious complications, decreased circulating lymphocytes, and increased plasma IL-10 concentration. Multivariate regression analysis indicated that the increased percentage of PD-L1-expressing CD14^+^ monocytes was an independent risk factor for infectious complications in AP. Area under the ROC curve analysis showed the combination of Acute Physiology and Chronic Health Evaluation II score and PD-L1 and HLA-DR expression in CD14^+^ monocytes had high accuracy in predicting infectious complications in patients with AP.

**Conclusions:**

The PD-1/PD-L1 system plays an essential role in the early immunosuppression of AP. PD-L1 expression in CD14^+^ monocytes may be a new marker for predicting risk of infectious complications in patients with AP.

## Background

Acute pancreatitis (AP) is a common disease with varied outcomes [1]. Most patients present with mild, self-limiting disease with low morbidity and mortality. Nevertheless, approximately 15–20% of patients will develop a severe form of the disease that has a poor prognosis [2]. Morbidity and mortality rates of patients in this group are high, mainly due to multiorgan failure in the first week and subsequent infectious complications (IC) [3]. It has been reported that more than 80% of the mortality occurs at the latter stages as a result of infections [4, 5]. Therefore, the early identification of patients at risk for IC would allow appropriate clinical management to reduce mortality.

Although little is known about the mechanisms responsible for the development of IC, it has been suggested that immunological impairment in patients during the early phase of AP may be linked to increased susceptibility to subsequent infections [6]. Both experimental and clinical studies suggest that patients with AP have depressed defenses against infection because of a defect in monocyte and lymphocyte signaling, which increases the risk of infection [7, 8]. In addition, a recent study showed that the shift of the Th1/Th2 balance toward Th2 responses during the course of AP leads to alterations of immune function [6, 9]. Thus, monitoring the immune status of patients with AP may help detect patients at risk for IC.

Programmed cell death 1 (PD-1), a coinhibitory molecule, belongs to the CD28 family and is expressed mainly in activated T cells, natural killer T cells, and myeloid cells [10]. Programmed cell death ligand 1 (PD-L1) is a PD-1 ligand that is expressed on antigen-presenting cells and hematopoietic cells. The PD-1/PD-L1 immune checkpoint is believed to play critical roles in suppressing the immune system and mediating evasion of host immune surveillance in infectious diseases and malignant tumors [11, 12]. In sepsis, the PD-1/PD-L1 system has been found to reduce bacterial clearance and thus has been deemed an important marker for assessing immune status [13]. However, whether PD-1/PD-L1 participates in the immunosuppression of AP remains unknown.

In this study, we explored PD-1 expression in CD4^+^ T cells and PD-L1 in CD14^+^ monocytes in the peripheral blood of patients with AP. The aim of the study was to determine whether these parameters can be used to assess immune status and predict IC in patients with AP.

## Methods

### Patient information, data collection, and definitions

The study protocol was approved by the Ruijin Hospital Ethics Committee of Shanghai Jiaotong University School of Medicine, China. Formal informed consent was obtained from patients or their next of kin. Between March 2014 and December 2015, 63 patients diagnosed with AP were recruited from the general intensive care unit (ICU) or emergency ICU of Ruijin Hospital; 32 healthy volunteers matched with sex and age were included as control subjects. AP diagnosis is most often established by the presence of two of the following three criteria: (1) abdominal pain consistent with the disease, (2) serum amylase and/or lipase greater than three times the normal upper limit, and/or (3) characteristic findings based on abdominal imaging [14]. The inclusion criteria were patients with AP aged 18 years or older who were admitted to the ICU within 24 h of symptom onset. Patients were excluded if any of the following criteria were present: suspected malignancy of the pancreas or biliary tree, a medical history of immunodeficiency or receiving immunosuppressive therapy, or nonpancreatic infection or sepsis caused by a second disease. All admissions were followed until discharge from the hospital or hospital mortality. Baseline characteristics, including age, sex, possible AP etiology, and Acute Physiology and Chronic Health Evaluation II (APACHE II) score were also collected and recorded.

AP severity was categorized as mild, moderately severe (local complication or transient organ dysfunction), or severe (persistent organ dysfunction) according to the revised Atlanta classification system [15]. Infected pancreatic necrosis, bacteremia, pneumonia, infected ascites, or urosepsis during admission and/or 90-day follow-up were considered IC [16]. Infected necrosis was defined as a positive culture of peripancreatic fluid or pancreatic necrosis obtained during the first percutaneous drainage or during the first surgical intervention. Bacteremia was defined by a positive blood culture sample. Pneumonia was defined by coughing, dyspnea, chest radiograph showing infiltrative abnormalities, and lowered arterial blood gas with positive sputum culture. Infected ascites was defined as a positive bacterial culture in aspirate of intraperitoneal fluid or abdominal fluid during surgical operation. Urosepsis was defined as dysuria with bacteremia on the same day. For patients without bacterial evidence, diagnosis of IC was made by experienced clinicians on the basis of clinical symptoms and signs of patients during admission and at 90-day follow-up. All infections were weighted equally; multiple infections in the same patient were considered one endpoint.

### Blood collection and processing

Peripheral venous blood samples were obtained from each patient and each healthy volunteer. Blood samples were transported to the clinical research center at 4 °C within 1 h. Plasma was obtained after centrifugation (3,000 *× g*, 10 minutes, 4 °C) and stored at −80 °C for further analysis.

### Flow cytometry and serum interleukin-10 analysis

Blood samples were obtained from 63 patients with AP at days 1 and 3 after diagnosis with AP and from 32 healthy volunteers. After lysing red blood cells with fluorescence-activated cell sorting lysing solution (BD Biosciences, San Jose, CA, USA), cells were incubated in the dark at 4 °C for 30 minutes with the following fluoresceinated monoclonal antibodies and their isotype controls: fluorescein isothiocyanate (FITC)-labeled anti-CD4 (clone A161A1), FITC-labeled anti-CD14 (clone HCD14), phycoerythrin (PE)-labeled anti-PD-1 (clone EH12.2H7), PE-labeled anti-PD-L1 (clone 29E.2A3), and PE-labeled anti-human leukocyte antigen-DR (anti-HLA-DR) (clone L243; BioLegend, San Diego, CA, USA). Stained cells were analyzed using a FACSCalibur flow cytometer and CellQuest software (BD Biosciences). The percentage of PD-1-expressing CD4^+^ lymphocytes was calculated as the percentage of PD-1^+^ cells in the total CD4^+^ lymphocyte population, and the percentage of PD-L1/HLA-DR-expressing CD14^+^ monocytes was calculated as the percentage of PD-L1^+^/HLA-DR^+^ cells in the total CD14^+^ monocyte population. Interleukin (IL)-10 concentration was measured by enzyme-linked immunosorbent assay (R&D Systems, Minneapolis, MN, USA) in accordance with the supplied instructions.

### Statistical analysis

Continuous variables are presented as mean ± SEM, and categorical variables are presented as absolute numbers and percentages. All variables were tested for normal distribution using the Kolmogorov-Smirnov test. Student’s *t* test was used to compare the means of continuous variables and the normality of data distribution; otherwise, the Mann-Whitney *U* test was used. Categorical data were tested using the χ^2^ test. Correlations between PD-1/PD-L1 expression, lymphocyte count, and IL-10 concentration were analyzed with Spearman’s rank method. Discrimination was tested using the area under the ROC curve (AUROC) to assess the ability of APACHE II score, PD-L1 expression in CD14^+^ monocytes, and HLA-DR expression in CD14^+^ monocytes to predict IC. To evaluate the predictive value of the combination of APACHE II score and HLA-DR and PD-L1 expression levels on monocytes, we constructed a predictive logistic regression model including the three variables. The coefficients for HLA-DR, PD-L1, and APACHE II score were −7.765, 9.867, and 0.323, respectively, and the constant was −3.723. On the basis of this model, we created a new variable using the formula [0.323 × APACHE II score −7.765 × PD-L1 + 9.867 × HLA-DR −3.723] to calculate the AUROC further. All statistical tests were two-tailed, and *P* < 0.05 was considered statistically significant. Statistical analyses were performed using STATA 12.0 for Windows software (StataCorp, College Station, TX, USA).

## Results

### Patient clinical characteristics

A total of 63 patients with AP (22 women and 41 men) were included in the study. Median age at admission was 48 years (IQR 37–60). The etiologies of AP were gallstones in 30 patients, hypertriglyceridemia in 20, alcohol abuse in 7, and other etiologies in 6. The median APACHE II score upon AP diagnosis was 10.5 (IQR 8–16.5), indicating a high level of disease severity. According to the revised Atlanta classification, 3 patients were classified as mild, 28 as moderately severe, and 32 as severe. The clinical characteristics of these patients are summarized in Table 1.

**Table 1:** Demographic and clinical data for patients with acute pancreatitis

Characteristics	Data (*n* = 63)
Age, years	48 (37 to 60)
Female/male, *n*	22/41
Etiology of acute pancreatitis, *n*	
Biliary	30 (47.6%)
Hypertriglyceridemia	20 (31.7%)
Alcohol	7 (11.1%)
Other	6 (9.5%)
APACHE II score	10.5 (8.0 to 16.3)
Severity of AP, *n* (%)	
Mild	3 (4.8%)
Moderately severe	28 (44.4%)
Severe	32 (50.8%)
Organ dysfunction	
Respiratory	31
Cardiovascular	10
Renal	11
Infection site	
Bacteremia	6
Pneumonia	6
Infected necrosis	19
Interventions	
Mechanical ventilation	22
CRRT	17
Surgical	8

*Abbreviations: AP* Acute pancreatitis, *APACHE* Acute Physiology and Chronic Health Evaluation, *CRRT* continuous renal replacement therapy

Values are presented as median and IQR for continuous variables or as number of cases and percent for categorical data

### PD-1 expression in CD4^+^ lymphocytes and PD-L1 expression in CD14^+^ monocytes from patients with AP

**Fig. 1:**
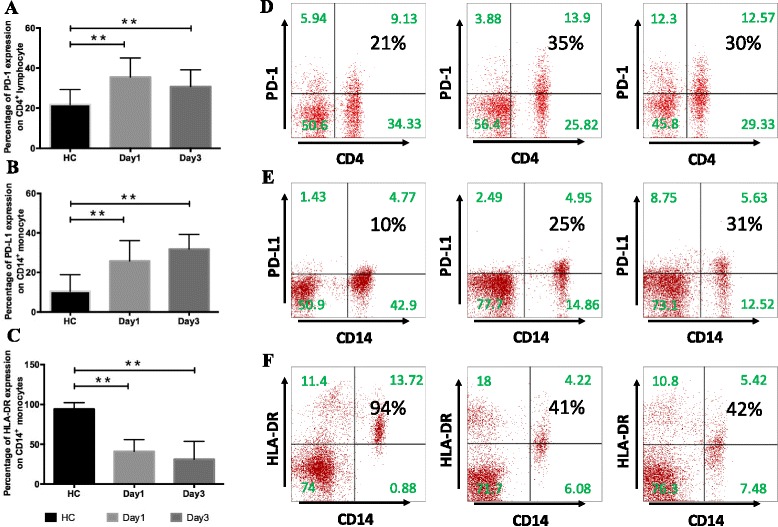
Programmed cell death 1 (PD-1) expression in CD4^+^ lymphocytes and programmed cell death ligand 1 (PD-L1) expression in CD14^+^ monocytes in patients with acute pancreatitis (AP). **a** PD-1 expression in circulating CD4^+^ lymphocytes and **b** PD-L1 and **c** human leukocyte antigen (HLA)-DR expression in CD14^+^ monocytes were measured in whole blood from healthy control subjects (HC, *n* = 32) and patients with AP (*n* = 63) on day 1 (D1) and day 3 (D3) after disease onset. Results are presented as percentages of positive cells among the total population of CD14^+^ monocytes or CD4^+^ lymphocytes. **d** Representative PD-1 expression levels in CD4^+^ lymphocytes and **e** PD-L1 and **f** HLA-DR expression in CD14^+^ monocytes. Values in the *upper right quadrant* indicate the percentage of cells that express PD-1, PD-L1, or HLA-DR. *P < 0.05 compared with healthy controls. **P < 0.01 compared with healthy controls

Both PD-1 expression in CD4^+^ lymphocytes and PD-L1 expression in CD14^+^ monocytes were measured in each patient on day 1 (D1) and day 3 (D3) after AP onset (Fig. 1). The percentage of PD-1-expressing CD4^+^ lymphocytes on D1 and D3 was significantly increased in patients with AP compared with healthy volunteers (D1 *P* < 0.001; D3 *P* < 0.001) (Fig. 1). Likewise, the percentage of PD-L1-expressing CD14^+^ monocytes on D1 and D3 was notably increased in patients with AP compared with healthy volunteers (D1 *P* < 0.001; D3 *P* < 0.001) (Fig. 1). In addition, the percentage of HLA-DR-expressing CD14^+^ monocytes at each time point was significantly lower in patients with AP than in healthy volunteers (D1 *P* < 0.001; D3 *P* < 0.001) (Fig. 1).

### Correlation between plasma IL-10 concentration, circulating lymphocyte count, and PD-1/PD-L1 expression

It has been reported that the PD-1/PD-L1 system is involved in lymphocyte apoptosis and IL-10 production in sepsis [12, 13]. Thus, we explored the correlation between plasma IL-10 concentration, circulating lymphocyte count, and PD-1/PD-L1 expression. We found that circulating lymphocyte counts were inversely correlated with the percentage of PD-1-expressing CD4^+^ lymphocytes on D1 and D3 (*r* = −0.252, *P* = 0.047; and *r* = −0.297, *P* = 0.018, respectively). A negative correlation was also observed between circulating lymphocyte count and the percentage of PD-L1-expressing CD14^+^ monocytes on D1 (*r* = −0.302, *P* = 0.016) but not on D3. Moreover, positive correlations were observed between IL-10 concentration and the percentage of PD-L1-expressing CD14^+^ monocytes on D1 and D3 (*r* = 0.296, *P* = 0.019; and *r* = 0.459, *P* < 0.001, respectively). The percentage of PD-1-expressing CD4^+^ lymphocytes on D1 was positively correlation with IL-10 concentration (*r* = 0.255, *P* = 0.044) but not on D3 (Table 2).

**Table 2:** Correlation between lymphocyte count, IL-10 concentration, and PD-1-related molecules

	Statistics	Lymphocyte count	IL-10 concentration
Percentage of PD-1 expression on CD4^+^ lymphocytes on day 1 (%)	*r*	−0.252	0.255
	*P*	0.047	0.044
Percentage of PD-1 expression on CD4^+^ lymphocytes on day 3 (%)	*r*	−0.297	0.125
	*P*	0.018	0.329
Percentage of PD-L1 expression on CD14^+^ monocytes on day 1 (%)	*r*	−0.302	0.296
	*P*	0.016	0.019
Percentage of PD-L1 expression on CD14^+^ monocytes on day 3 (%)	*r*	−0.012	0.459
	*P*	0.927	<0.001

*Abbreviations: PD-1* Programmed cell death receptor-1, *PD-L1* programmed cell death receptor ligand-1, *IL-10* Interleukin 10

### PD-1-expressing CD4^+^ lymphocytes and PD-L1-expressing CD14^+^ monocytes in patients with AP with or without IC

To assess the association between PD-1/PD-L1 expression and IC, patients were divided into two groups according to the presence of IC, namely IC (patients with IC, *n* = 25) and non-IC (patients without IC, *n* = 38) groups. In the patients with IC, 24% had bloodstream infection, 24% had pneumonia, and 79.2% had infected (peri)pancreatic necrosis (Table 1). There were no significant differences between these two groups in regard to sex (*P* > 0.05) (Table 3). Patients in the IC group were significantly older than those in the non-IC group. The APACHE II scores in the IC group were significantly higher than in the non-IC group (*P* < 0.05). Patients with IC showed significantly greater PD-1 expression in CD4^+^ T cells on D1 and D3 than patients without IC (D1 *P* < 0.05; D3 *P* < 0.05) (Table 3). Likewise, the percentage of PD-L1-expressing CD14^+^ monocytes on D1 and D3 was higher in the IC group than in the non-IC group (D1 *P* < 0.05; D3 *P* < 0.05) (Table 3).

**Table 3:** Clinical characteristics of patients with acute pancreatitis with or without infectious complications

	Non-IC group (*n* = 38)	IC group (*n* = 25)	*p* Value
Age, years	45.34 ± 2.56	53.60 ± 2.53	0.037
Female/male, *n*	14/24	8/17	
WBC count, ×10^9^/L	12.49 ± 0.93	13.32 ± 1.06	0.559
BUN, mmol/L	12.71 ± 1.26	17.22 ± 2.49	0.088
Hematocrit	0.41 ± 0.01	0.42 ± 0.02	0.701
APACHE II score	10 ± 0.68	15.12 ± 1.10	0.000
Lymphocyte count on day 1, ×10^9^/L	1.00 ± 0.05	0.79 ± 0.05	0.012
Lymphocyte count on day 3, ×10^9^/L	1.08 ± 0.06	0.86 ± 0.06	0.016
IL-10 concentration on day 1, pg/ml	30.83 ± 1.56	41.86 ± 2.56	0.000
IL-10 concentration on day 3, pg/ml	26.09 ± 1.37	32.85 ± 2.24	0.017
Percentage of HLA-DR expression on CD14^+^ monocytes on day 1	44.51 ± 2.19	36.30 ± 2.99	0.027
Percentage of HLA-DR expression on CD14^+^ monocytes on day 3	46.64 ± 2.01	37.24 ± 2.86	0.010
Percentage of PD-1 expression on CD4^+^ lymphocytes on day 1, (%)	33.31 ± 1.40	38.83 ± 2.02	0.024
Percentage of PD-1 expression on CD4^+^ lymphocytes on day 3	28.98 ± 1.13	33.38 ± 1.95	0.041
Percentage of PD-L1 expression on CD14^+^ monocytes on day 1	22.96 ± 1.42	29.94 ± 2.26	0.010
Percentage of PD-L1 expression on CD14^+^ monocytes on day 3	30.00 ± 1.04	34.46 ± 1.67	0.020
Mortality	2 (9.09%)	2 (6.67%)	1.000

*Abbreviations: APACHE* Acute Physiology and Chronic Health Evaluation, *BUN* Blood urea nitrogen, *IL* Interleukin, *HLA-DR* Human leukocyte antigen-DR, *IC* Infectious complications, *PD-1* Programmed cell death receptor 1, *PD-L1* Programmed cell death receptor ligand 1, *WBC* White blood cell

Data are shown as mean ± SEM

Moreover, biomarkers commonly used to assess immune status were measured, and we found that the circulating lymphocyte counts and percentage of HLA-DR-expressing CD14^+^ monocytes on D1 and D3 were decreased in the IC group compared with the non-IC group. Additionally, levels of the anti-inflammatory cytokine IL-10 in the IC group were increased compared with the non-IC group (Table 3).

### Value of PD-L1 expression in CD14^+^ monocytes on day 1 for predicting IC in patients with AP

Univariate logistic regression analysis (Table 4) was performed to determine the predictive power of age, APACHE II score at admission, lymphocyte count on D1, percentage of HLA-DR-expressing CD14^+^ monocytes on D1, percentage of PD-1-expressing CD4^+^ lymphocytes on D1, and percentage of PD-L1-expressing CD14^+^ monocytes on D1 for IC. The variables showed significant predictive value within univariate analysis and were included in further stepwise multivariate logistic regression (Table 4). The regression analysis detected APACHE II score at admission (OR 1.305, 95% CI 1.079–1.578, *P* = 0.006), percentage of HLA-DR-expressing CD14^+^ monocytes on D1 (OR 0.923, 95% CI 0.869–0.981, *P* = 0.01), and percentage of PD-L1-expressing CD14^+^ monocytes on D1 (OR 1.098, 95% CI 1.005–1.200, *P* = 0.036) as independent significant predictors of IC in patients with AP.

**Table 4:** Univariate and multivariate logistic regression analysis for infectious complications

Elements	Univariate analysis			Multivariate analysis		
	OR	95% CI	*p* Value	OR	95% CI	*p* Value
Age	1.038	1.001–1.077	0.041	1.025	0.969–1.084	0.386
APACHE II score	1.249	1.097–1.422	0.001	1.305	1.079–1.578	0.006
Lymphocyte count on day 1 × 10^9^/L	0.821	0.691–0.975	0.025	0.956	0.697–0.761	0.691
Percentage of HLA-DR expression on CD14^+^ monocytes on day 1	0.959	0.923–0.996	0.031	0.923	0.869–0.981	0.01
Percentage of PD-1 expression on CD4^+^ lymphocytes on day 1	1.070	1.006–1.137	0.031	1.046	0.956–1.143	0.327
Percentage of PD-L1 expression on CD14^+^ monocytes on day 1	1.075	1.016–1.138	0.013	1.098	1.005–1.200	0.036

*Abbreviations: APACHE* Acute Physiology and Chronic Health Evaluation, *HLA-DR* Human leukocyte antigen-DR, *PD-1* Programmed cell death receptor 1, *PD-L1* Programmed cell death receptor ligand 1

**Fig. 2:**
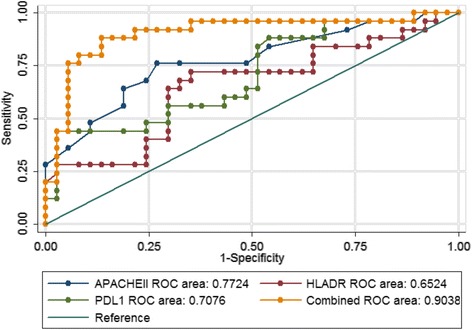
Receive operating characteristic (ROC) curve for predicting infectious complications in patients with AP. *APACHE* Acute Physiology and Chronic Health Evaluation, *HLA-DR* Human leukocyte antigen-DR, *PD-L1* Programmed cell death receptor ligand-1

The predictive accuracy value for IC in AP was determined by ROC curve analysis (Table 5, Fig. 2). The AUROC values were 0.772 (95% CI 0.649–0.896, *P* < 0.01) for APACHE II score at admission, 0.652 (95% CI 0.506–0.798, *P* = 0.043) for percentage of HLA-DR-expressing CD14^+^ monocytes on D1, and 0.708 (95% CI 0.573–0.842, *P* < 0.01) for percentage of PD-L1-expressing CD14^+^ monocytes on D1. The combination of these variables had a moderate to high accuracy for predicting IC (95% CI 0.816–0.991, *P* < 0.01). Table 5 shows the chosen cutoff points, sensitivity, specificity, and positive and negative predictive values of these variables for predicting development of IC.

**Table 5:** AUCs of various parameters for predicting infectious complications in patients with acute pancreatitis

Element	AUC	*p* Value	95% CI	
			Lower limit	Upper limit
APACHE II score	0.772	<0.01	0.649	0.896
Percentage of HLA-DR expression on CD14^+^ monocytes on day 1, (%)	0.652	0.043	0.506	0.798
Percentage of PD-L1 expression on CD14^+^ monocytes on day 1, (%)	0.708	<0.01	0.573	0.842
Combination of above three variables	0.904	<0.01	0.816	0.991

*Abbreviations: APACHE* Acute Physiology and Chronic Health Evaluation, *HLA-DR* Human leukocyte antigen-DR, *PD-L1* Programmed cell death receptor ligand 1

## Discussion

The development of IC determines hospital stay and prognosis for patients with AP [3]. Therefore, early detection of IC may enable clinicians to timely and appropriately treat patients. Clinically, there is an urgent need to identify a marker that is predictive of IC during the early stage of AP. In this study, we found increased expression of PD-1 in CD4^+^ lymphocytes and PD-L1 in CD14^+^ monocytes in patients with AP, especially those with IC. PD-1 expression in CD4^+^ lymphocytes and PD-L1 expression in CD14^+^ monocytes correlated to peripheral lymphocyte count and plasma IL-10 level, which indicated that the PD-1/PD-L1 system takes part in the development of immunosuppression in AP. The percentages of PD-L1-expressing CD14^+^ monocytes and HLA-DR-expressing CD14^+^ monocytes on D1 and APACHE II score upon admission were independently associated with IC in AP. Additionally, the combination of these variables could predict the development of IC with high accuracy in patients with AP.

Numerous studies have demonstrated that immunosuppression is a critically important risk factor for IC in AP [6]. Recently, the PD-1/PD-L1 system was reported to be involved in immunosuppressive mechanisms [10]. During the progression of AP, a great number of inflammatory cytokines are boosted, and the inflammatory cytokines, such as tumor necrosis factor-α, can then induce PD-1 and PD-L1 expression [10, 17]. Originally described as an apoptotic factor, PD-1/PD-L1 has been reported to regulate the proliferative capacity and apoptosis of lymphocytes [18, 19]. It is possible that PD-1/PD-L1 stimulation can direct cells into a G_0_ resting state, inhibiting lymphocyte proliferation [18, 20]. Furthermore, the PD-1/PD-L1 system was also reported to induce IL-10 production [21], which then acts synergistically with PD-1/PD-L1 signaling to suppress T-cell responses [22]. In our study, PD-1 expression in CD4^+^ lymphocytes and PD-L1 expression in CD14^+^ monocytes were correlated with peripheral lymphocyte count and plasma IL-10 concentration, suggesting that the PD-1/PD-L1 system takes part in the development of immunosuppression in AP by regulating lymphocyte proliferation and IL-10 production.

Increased PD-1 and PD-L1 expression were associated with increased occurrence of secondary nosocomial infections in patients with septic shock [13]. In our study, PD-1 expression in CD4^+^ lymphocytes and PD-L1 expression in CD14^+^ monocytes were increased in patients with AP, especially those with IC. Moreover, the percentage of PD-L1-expressing CD14^+^ monocytes was independently associated with IC in AP rather than with PD-1 expression in CD4^+^ lymphocytes. This may be due to the fact that PD-L1 plays a major role in the PD-1/PD-L1 pathway [23], and PD-L1 is expressed more widely in immune and parenchymal tissue cells. Postmortem studies have demonstrated that PD-L1 is highly expressed in parenchymal tissue cells (i.e., splenic endothelial and bronchial epithelial cells), thereby providing an opportunity for PD-1 activation [24]. In that sense, PD-L1 expression in CD14^+^ monocytes not only may play a role in immune dysfunction but also may be an early indicator of IC in AP. Conversely, preoperative PD-1 expression in CD4^+^ lymphocytes was found to be independently associated with postoperative IC [25]. This discrepancy may be attributed to different pathophysiologies of the patient cohorts.

The hallmark of immunosuppression is monocyte deactivation, which is characterized by decreased HLA-DR expression and circulating lymphocyte counts [26, 27]. We found decreased circulating lymphocyte counts and HLA-DR expression in CD14^+^ monocytes in patients with IC compared with those without IC, which is consistent with the phenomenon of immune dysfunction. Shen et al. recently demonstrated that reduced lymphocyte count within 48 h of AP onset is independently associated with the development of infected pancreatic necrosis [28]. However, we found differences only in circulating lymphocyte counts between the IC and non-IC groups; lymphocyte count on D1 was not an independent factor for IC in AP after multivariate regression analysis. This needs to be investigated further in studies with larger sample sizes. HLA-DR is a common immunological marker that is evaluated in many hospitals. We found that the percentage of HLA-DR-expressing CD14^+^ monocytes on D1 was independently associated with IC in AP. In many studies, HLA-DR expression was demonstrated to be closely related to poor prognosis in patients with AP [29, 30]. However, this parameter did not better predict IC in comparison with the percentage of PD-L1-expressing CD14^+^ monocytes on D1 in our study. This too needs to be further verified in other prospective studies.

Another novel finding of our study was that the combination of APACHE II score upon admission and D1 percentages of HLA-DR-expressing CD14^+^ monocytes and PD-L1-expressing CD14^+^ monocytes could improve the accuracy of predicting IC in patients with AP. Immune status can be influenced by many factors, including HLA-DR and PD-L1 expression in monocytes, and the APACHE II scoring system encompasses clinical parameters to grade disease severity. The combination of clinical risk factors and immune markers could be the optimal methodology to predict development of IC in patients with AP.

There are several limitations associated with this study. First, we studied these markers in patients admitted to the ICU; therefore, our results may not be generalizable to patients with less severe illness. Second, the number of patients studied was small, and future studies with larger cohorts are needed to validate these results.

## Conclusions

We describe the novel finding that expression of PD-1 in CD4^+^ lymphocytes and PD-L1 in CD14^+^ monocytes was markedly increased in patients with AP. Moreover, increased PD-1 and PD-L1 expression appears to be correlated with the development of immune dysfunction and IC. Expression of PD-L1 in CD14^+^ monocytes is independently associated with IC in AP.

## Abbreviations

AP: Acute pancreatitis
APACHE: Acute Physiology and Chronic Health Evaluation
AUROC: Area under the ROC curve
BUN: Blood urea nitrogen
CRRT: continuous renal replacement therapy
FITC: Fluorescein isothiocyanate
HC: Healthy control subject
HLA-DR: Human leukocyte antigen-DR
IC: Infectious complications
ICU: Intensive care unit
IL: Interleukin
PD-1: Programmed cell death 1
PD-L1: Programmed cell death ligand 1
PE: phycoerythrin

## References

[1] De Waele JJ (2014). Acute pancreatitis. Curr Opin Crit Care.

[2] Gomatos IP, Xiaodong X, Ghaneh P, Halloran C, Raraty M, Lane B (2014). Prognostic markers in acute pancreatitis. Expert Rev Mol Diagn.

[3] Banks PA, Freeman ML (2006). Practice guidelines in acute pancreatitis. Am J Gastroenterol.

[4] Buddingh KT, Koudstaal LG, van Santvoort HC, Besselink MG, Timmer R, Rosman C (2014). Early angiopoietin-2 levels after onset predict the advent of severe pancreatitis, multiple organ failure, and infectious complications in patients with acute pancreatitis. J Am Coll Surg.

[5] Gloor B, Muller CA, Worni M, Martignoni ME, Uhl W, Buchler MW (2001). Late mortality in patients with severe acute pancreatitis. Br J Surg.

[6] Li JP, Yang J, Huang JR, Jiang DL, Zhang F, Liu MF (2013). Immunosuppression and the infection in patients with early SAP. Front Biosci (Landmark Ed).

[7] Oiva J, Mustonen H, Kylanpaa ML, Kyhala L, Kuuliala K, Siitonen S (2010). Acute pancreatitis with organ dysfunction associates with abnormal blood lymphocyte signaling: controlled laboratory study. Crit Care.

[8] Oiva J, Mustonen H, Kylänpää ML, Kyhälä L, Alanärä T, Aittomäki S (2010). Patients with acute pancreatitis complicated by organ failure show highly aberrant monocyte signaling profiles assessed by phospho-specific flow cytometry. Crit Care Med.

[9] Pietruczuk M, Dabrowska MI, Wereszczynska-Siemiatkowska U, Dabrowski A (2006). Alteration of peripheral blood lymphocyte subsets in acute pancreatitis. World J Gastroenterol.

[10] Keir ME, Butte MJ, Freeman GJ, Sharpe AH (2008). PD-1 and its ligands in tolerance and immunity. Annu Rev Immunol..

[11] Butte MJ, Keir ME, Phamduy TB, Sharpe AH, Freeman GJ (2007). Programmed death-1 ligand 1 interacts specifically with the B7-1 costimulatory molecule to inhibit T cell responses. Immunity.

[12] Yokoyama S, Miyoshi H, Nakashima K, Shimono J, Hashiguchi T, Mitsuoka M (2016). Prognostic value of programmed death ligand 1 and programmed death 1 expression in thymic carcinoma. Clin Cancer Res.

[13] Guignant C, Lepape A, Huang X, Kherouf H, Denis L, Poitevin F (2011). Programmed death-1 levels correlate with increased mortality, nosocomial infection and immune dysfunctions in septic shock patients. Crit Care.

[14] Tenner S, Baillie J, DeWitt J, Vege SS (2013). American College of Gastroenterology guideline: management of acute pancreatitis. Am J Gastroenterol.

[15] Banks PA, Bollen TL, Dervenis C, Gooszen HG, Johnson CD, Sarr MG (2013). Classification of acute pancreatitis—2012: revision of the Atlanta classification and definitions by international consensus. Gut.

[16] Besselink MG, van Santvoort HC, Buskens E, Boermeester MA, van Goor H, Timmerman HM (2008). Probiotic prophylaxis in predicted severe acute pancreatitis: a randomised, double-blind, placebo-controlled trial. Lancet.

[17] Abiko K, Matsumura N, Hamanishi J, Horikawa N, Murakami R, Yamaguchi K (2015). IFN-γ from lymphocytes induces PD-L1 expression and promotes progression of ovarian cancer. Br J Cancer.

[18] Chang K, Svabek C, Vazquez-Guillamet C, Sato B, Rasche D, Wilson S (2014). Targeting the programmed cell death 1: programmed cell death ligand 1 pathway reverses T cell exhaustion in patients with sepsis. Crit Care.

[19] Zhang Y, Zhou Y, Lou J, Li J, Bo L, Zhu K (2010). PD-L1 blockade improves survival in experimental sepsis by inhibiting lymphocyte apoptosis and reversing monocyte dysfunction. Crit Care.

[20] Latchman Y, Wood CR, Chernova T, Chaudhary D, Borde M, Chernova I (2001). PD-L2 is a second ligand for PD-1 and inhibits T cell activation. Nat Immunol.

[21] Said EA, Dupuy FP, Trautmann L, Zhang Y, Shi Y, El-Far M (2010). Programmed death-1-induced interleukin-10 production by monocytes impairs CD4^+^ T cell activation during HIV infection. Nat Med.

[22] Huang A, Zhang B, Yan W, Wang B, Wei H, Zhang F (2014). Myeloid-derived suppressor cells regulate immune response in patients with chronic hepatitis B virus infection through PD-1-induced IL-10. J Immunol.

[23] Shao R, Fang Y, Yu H, Zhao L, Jiang Z, Li CS (2016). Monocyte programmed death ligand-1 expression after 3-4 days of sepsis is associated with risk stratification and mortality in septic patients: a prospective cohort study. Crit Care.

[24] Boomer JS, To K, Chang KC, Takasu O, Osborne DF, Walton AH (2011). Immunosuppression in patients who die of sepsis and multiple organ failure. JAMA.

[25] Kubo T, Ono S, Miyazaki H, Saitoh D, Yamamoto J, Hase K (2015). Perioperative programmed death 1 expression on CD4^+^ T cells predicts the incidence of postoperative infectious complications. Shock.

[26] Bone RC (1996). Sir Isaac Newton, sepsis, SIRS, and CARS. Crit Care Med.

[27] Volk HD, Waschke SR, Diezel W, Grunow R, von Baehr R, Fiebig H (1985). Decrease of HLA-DR antigen expression by human monocytes during cultivation in absence of exogenous or endogenous interferon-γ. Immunol Lett.

[28] Shen X, Sun J, Ke L, Zou L, Li B, Tong Z (2015). Reduced lymphocyte count as an early marker for predicting infected pancreatic necrosis. BMC Gastroenterol..

[29] Ho YP, Sheen IS, Chiu CT, Wu CS, Lin CY (2006). A strong association between down-regulation of HLA-DR expression and the late mortality in patients with severe acute pancreatitis. Am J Gastroenterol.

[30] Sachse C, Prigge M, Cramer G, Pallua N, Henkel E (1999). Association between reduced human leukocyte antigen (HLA)-DR expression on blood monocytes and increased plasma level of interleukin-10 in patients with severe burns. Clin Chem Lab Med.

